# Genome-Wide Screening for Genes Associated with Valproic Acid Sensitivity in Fission Yeast

**DOI:** 10.1371/journal.pone.0068738

**Published:** 2013-07-05

**Authors:** Lili Zhang, Ning Ma, Qingbin Liu, Yan Ma

**Affiliations:** Division of Molecular Pharmacology and Pharmacogenomics, Department of Biochemistry and Molecular Biology, Kobe University Graduate School of Medicine, Kobe, Japan; University of Cambridge, United Kingdom

## Abstract

We have been studying the action mechanisms of valproic acid (VPA) in fission yeast *Schizosaccharomyces pombe* by developing a genetic screen for mutants that show hypersensitivity to VPA. In the present study, we performed a genome-wide screen of 3004 haploid deletion strains and confirmed 148 deletion strains to be VPA sensitive. Of the 148 strains, 93 strains also showed sensitivity to another aliphatic acids HDAC inhibitor, sodium butyrate (SB), and 55 strains showed sensitivity to VPA but not to SB. Interestingly, we found that both VPA and SB treatment induced a marked increase in the transcription activity of Atf1 in wild-type cells. However, in *clr6-1*, a mutant allele the *clr6^+^* gene encoding class I HDAC, neither VPA- nor SB induced the activation of Atf1 transcription activity. We also found that VPA, but not SB, caused an increase in cytoplasmic Ca^2+^ level. We further found that the cytoplasmic Ca^2+^ increase was caused by Ca^2+^ influx from extracellular medium via Cch1-Yam8 channel complex. Altogether, our present study indicates that VPA and SB play similar but distinct roles in multiple physiological processes in fission yeast.

## Introduction

VPA is a short-chain branched fatty acid that was discovered serendipitously as an anticonvulsant while being used as a solvent. Today VPA is used to treat a variety of psychiatric diseases such as seizure disorders, bipolar disorder and migraine [1], [2], that is supposed by targeting GABA transaminase, succinate semialdehyde dehydrogenase, and alpha-ketoglutarate dehydrogenase and Na^+^ channels [3], [4]. In 2001, histone deacetylases (HDACs) were identified as direct targets of VPA [5], [6]. In mammalian cells, ten structurally related HDACs have been classified into three classes: class I (HDAC1, 2, 3, and 8), class II (HDAC4, 5, 6, 7, 9, and 10) and class III (homologues of the yeast Sir2 proteins). The activity of class I and class II HDACs is inhibited by short chain fatty acids and hydroxamic acids, but class III HDACs are not inhibited by these agents [7]. HDACs catalyze the removal of the acetyl modification on lysine residues of histones, which is associated with a condensed chromatin structure resulting in the repression of gene transcription. HDAC inhibitors induce an increase in histone acetylation, which is associated with a loose chromatin structure thereby activating the transcription [8], [9]. As one of HDAC inhibitors, VPA has shown potent antitumor effects in a variety of *in vitro* and *in vivo* systems, and encouraging results in clinical trials either alone or in combination with demethylating and/or cytotoxic agents [10]. In addition, to its anti-cancer activity, HDAC inhibitors could benefit diseases such as neurological and psychiatric disorders [11]. Notably, although histone proteins were the first and the most important targets of HDACs, increasing evidence indicates that HDACs also deacetylate other nonhistone proteins such as α-tubulin, β-catenin, protein 53, 90-kDa heat shock protein, and et al [12]. These increasing studies suggest that the effects of HDAC inhibitors are likely to be much broader and more complicated than originally envisioned.

To better understand the molecular mechanisms of VPA action, we previously developed a genetic screen for mutants that show hypersensitivity to VPA. This genetic screen for mutants led us to the identification of *vas1^+^*/*vps45^+^* gene which encodes a member of the Sec1/Munc18 family [13], and *vas2^+^*/*aps1^+^* gene which encodes the σ subunit of AP-1 complex [14], and *vas3^+^*/*ric1^+^* gene which encodes guanyl-nucleotide exchange factor for Rab GTPase Ryh1 [15]. Our previous studies suggest that VPA affects membrane trafficking which leads to the enhanced sensitivity to cell-wall damage in fission yeast [13].

In the present study, we performed a genome-wide screen by using VPA and sodium butyrate (SB), another aliphatic acids HDAC inhibitor that effectively inhibits cell proliferation by cell cycle arrest and induces apoptosis by increasing histone hyperacetylation [16] [17]. Our results suggest that VPA and SB affect a variety of complex physiological processes such as DNA and RNA metabolism, signal transduction, membrane trafficking, chromatin remodeling, mitochondrial function, ubiquitination, transcription, genes encoding transporters, ribosomal protein and a variety of other well-known functions or still unknown functions in the biological system in fission yeast. Furthermore, we found that both VPA and SB treatment increased the transcription activity of Atf1 in wild-type cells, but not in *clr6-1* mutants, suggesting that VPA and SB increase the Atf1 transcriptional activity in a Clr6-dependent manner. Moreover, we also found VPA, but not SB, caused Ca^2+^ influx via the Cch1-Yam8 channel complex.

## Materials and Methods

### Strains, Media, Genetic and Molecular Biology Methods

Heterozygous diploid deletion strains were constructed by Bioneer Corporation and Korea Research Institute of Biotechnology and Bioscience (http://pombe.bioneer.co.kr/). These deletion strains were generated with a genetic background of h^+^/h^+^ *ade6-M210*/*ade6-M216 leu1-32*/*leu1-32 ura4-D18*/*ura4-D18* using PCR-based deletion method [18]. The haploid deletion library used in this study consists of 3004 nonessential genes, each of which carries a defined deletion of a characterized or a putative nonessential open reading frame replaced with the *kanMX4* cassette. Deletion of the target open reading frame was screened by G418 antibiotic selection. The other strains used in this study are listed in Table 1. The complete medium YPD (yeast extract-peptone-dextrose) and the minimal medium EMM (Edinburgh minimal medium) have been described previously [19]. YPD plates are supplemented with 225 mg/l adenine to produce YPDA (yeast peptone dextrose adenine) plates. Gene disruptions are abbreviated by the gene preceded by Δ (for example, Δ*cch1*). Proteins are denoted by Roman letters and only the first letter is capitalized (for example, Cch1).

**Table 1:** Strains used in this study.

Strain	Genotype	Reference
HM123	*h^−^ leu1-32*	Our stock
KP208	*h^−^ leu1-32 ura4-D18 pmk1*::*ura4^+^*	[46]
KP2758	*h^−^ leu1-32 ura4-D18 yam8*::*ura4^+^*	[35]
KP2784	*h^−^ leu1-32 ura4-D18 cch1*::*ura4^+^*	[35]
KP5967	*h^−^ leu1-32 clr6-1*	[47]
KP452	*h^−^ leu1-32 ura4-D18 mkh1*::*ura4^+^*	Our stock
KP2163	*h^−^ leu1-32 pck2*::*kanMX6*	Our stock
KP119	*h^+^ leu1-32 ura4-D18 ppb1*::*ura4^+^*	Our stock
KP495	*h^−^ leu1-32 ura4-D18 atf1*::*ura4^+^*	[48]

### Genome-wide Screen for VPA- and SB-Sensitive Deletion Mutants

We used streak method for single-colony isolation, a classical experimental approach that widely used for the study of yeast cells, to screen VPA- or SB-sensitive strains. The deletion mutant library was frozen at −80°C in 96-well microtitre plates in 30% glycerol in liquid YES medium. Prior to performing the experiment, the library was transferred to YES plates at 27°C. SB was purchased from Alfa Aesar, dissolved in distilled water to produce a stock solution of 1 M. The stock solution of VPA was produced as described previously [13]. VPA or SB was added to YPDA plates subsequent to autoclaving and cooling of the medium to 55°C. The log-phase cells were streaked onto YPDA plates with or without VPA or SB and incubated at 27°C for 6 days. Altered sensitivity was assessed by analyzing the size of the isolated colonies formed on each plate. We repeated the streak four times with good consistency.

### Miscellaneous Methods

The real-time monitoring of Atf1 transcriptional activity using the firefly luciferase reporter was measured as described previously [20]. Real-time monitoring of the cytoplasmic Ca^2+^ level using GFP-19-AEQ was measured as described previously [21]. Database searches were performed using the Sanger Center *S. pombe* database search service (www.sanger.ac.uk). Cell extract preparation and immunoblot analysis were performed as described [22]. TSA was purchased from Tokyo Chemical Industry Co., dissolved in ethyl alcohol to produce a stock solution of 10 µg/ml. Acetyl-histone H4 antibody set (Ac K5; Ac K8; Ac K12) were purchased from Upstate (Millipore). Anti-histone H4 antibody was purchased from abcam.

## Results and Discussion

### Identification of Genes Required for Growth on Valproic Acid (VPA) or Sodium Butyrate (SB) Containing Plates

**Figure 1:**
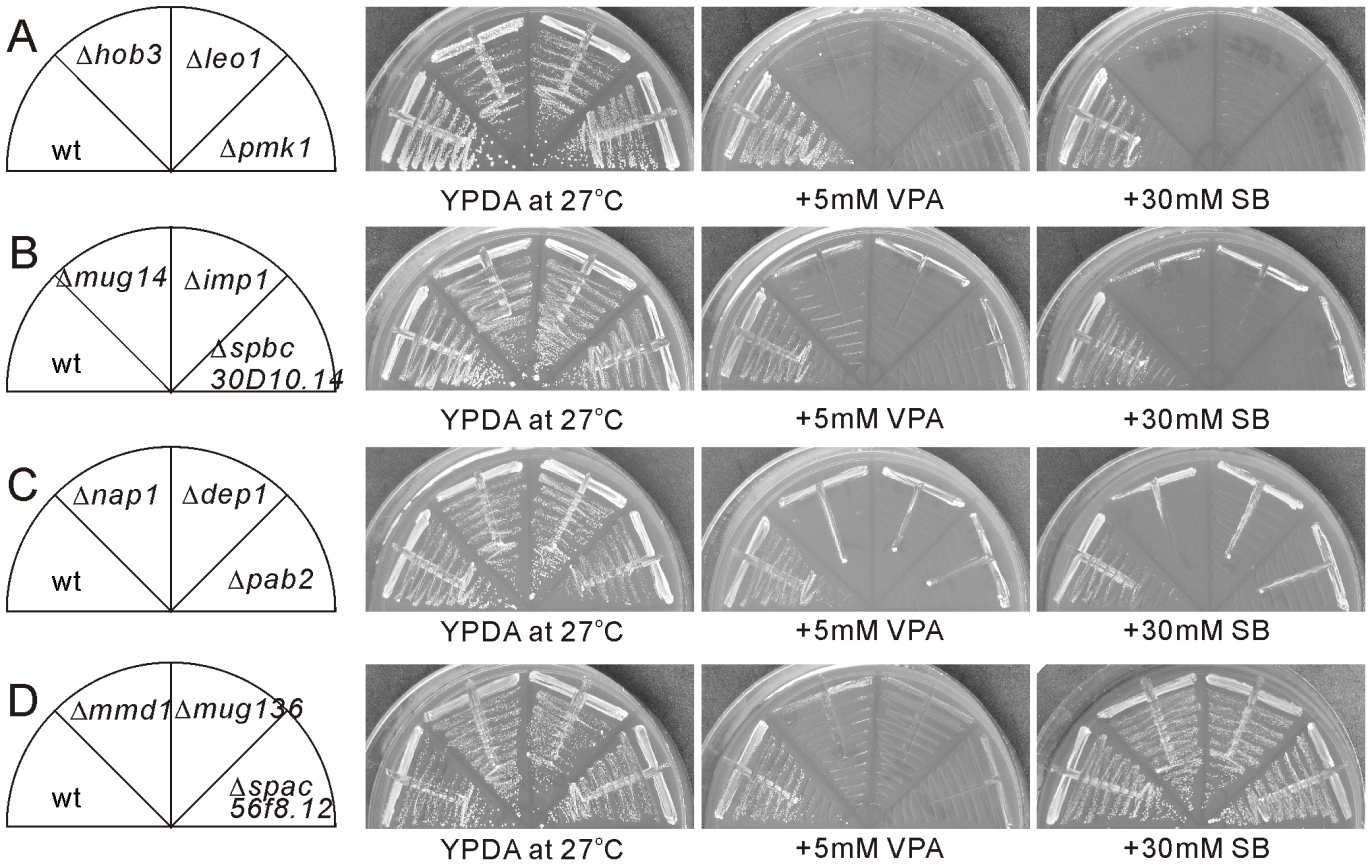
Representative growth pattern of the *S. pombe* deletion mutants in the presence of VPA or SB. The log-phase wild-type (wt) and deletion cells as indicated were streaked onto YPDA plates with or without 5 mM VPA or 30 mM SB, and incubated at 27°C for 6 days.

**Figure 2:**
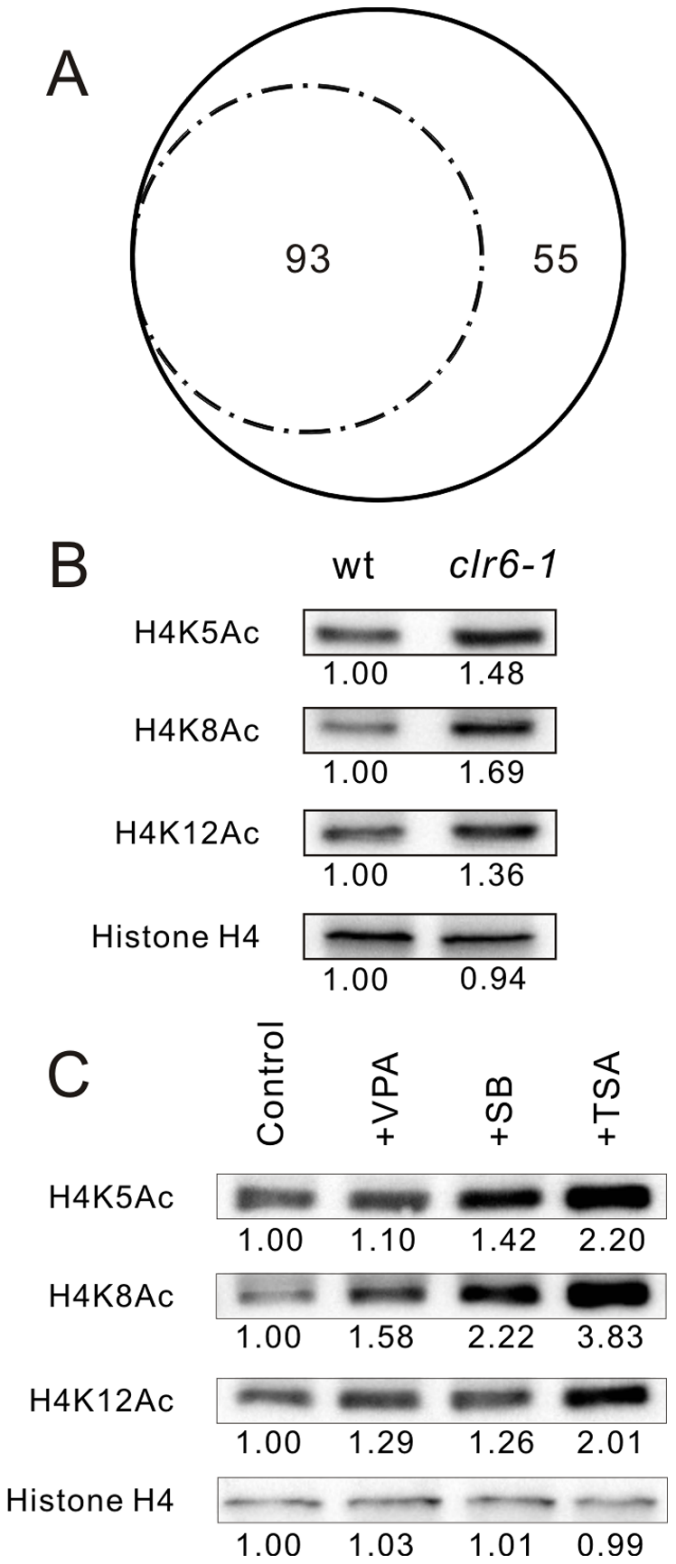
VPA and SB may function as HDAC inhibitor in fission yeast. (A) The Venn diagrams of VPA- or SB- sensitive strains. The SB-sensitive strains were completely included in VPA-sensitive strains. Of the 148 VPA-sensitive strains, 93 strains also showed sensitivity to SB, the other 55 strains only showed sensitivity to VPA. (B) Immunoblot analysis of histone acetylation. The wild-type cells and *clr6-1* mutants were cultured in YPD at 27°C for 10 hours to exponential phase. Then the cell extracts were subjected to electrophoresis using 11% polyacrylamide gel and were immunoblotted using Acetyl-histone H4 antibody set (Ac K5; Ac K8; Ac K12) to detect histone H4 acetylation. Endogenous levels of total histone H4 protein was used as a loading control and was immunoblotted using anti-histone H4 antibodies. (C) VPA and SB treatment increased histone H4 acetylation. The exponentially growing wild-type cells were divided into three equal portions. One portion is left without treatment and the other three portions were treated with 4 mM VPA, 60 mM SB or 20 µg/ml TSA for 20 minutes, respectively. Then the protein is extracted and immunoblotted as described in Figure 2A.

The yeast deletion collection is a powerful tool for identifying genes that are involved in drugs resistance and heavy metal resistance [23]–[28]. HDAC inhibitors are divided into several structural classes including hydroximates, cyclic peptides, aliphatic acids, and benzamides [29]–[31]. Both VPA and SB are aliphatic acids, and are recently shown to function as HDAC inhibitors [31]. To identify nonessential genes associated with increased sensitivity to VPA or SB, we compared colony sizes of wild-type cells with that of 3004 deletion cells by streaking on YPDA containing 5 mM VPA or 30 mM SB plates. The severity of growth inhibition by drugs was scored as follows: severe (+++) indicating that the cells completely failed to grow on the VPA- or SB-containing plates (Figure 1A), moderate (++) indicating that tiny colonies were observed to grow on the VPA- or SB-containing plates (Figure 1B), and mild (+) indicating that colonies were observed to grow on the VPA- or SB-containing plates however the colonies were significantly smaller than that of wild-type cells (Figure 1C). We confirmed 93 deletion strains displayed varying levels of sensitivities to both VPA and SB (Figure 1A-C and Table S1), and 55 deletion strains displayed varying levels of sensitivity to VPA only (Figure 1D and Table S1). We repeated the streak four times with good consistency. The number of SB- and VPA-sensitive strains and VPA-sensitive strains are counted in Venn diagrams (Figure 2A). The SB-sensitive strains were completely included in VPA-sensitive strains, suggesting that VPA may affect much broader pathways compared with SB in fission yeast. We additionally examined the effect of another HDAC inhibitor Trichostatin A (TSA) on growth inhibition in the 148 VPA-sensitive mutants. The severity of growth inhibition by 20 µg/ml TSA was scored and summarized in Table S1.

Among the total 148 VPA- or SB-sensitive mutants, 37 mutants were severely sensitive (+++), 24 mutants were moderately sensitive (++), and 87 mutants were mildly sensitive (+). Among the 93 SB-sensitive mutants, 21 mutants were severely sensitive (+++), 40 mutants were moderately sensitive (++), and 32 mutants were mildly sensitive (+). The 148 genes were grouped according to their functions. The largest group consisted of genes involved in DNA and RNA metabolism (24/148 = 16.2%), the second group consisted of genes involved in signal transduction (19/148 = 12.8%), the third and fourth groups consisted of genes involved in membrane trafficking (17/148 = 11.5%), and chromatin remodeling (15/148 = 10.1%), respectively. Other groups consisted of genes involved in mitochondrial function (11/148 = 7.4%), ubiquitination (10/148 = 6.8%), transcription (8/148 = 5.4%), genes encoding transporters (5/148 = 3.4%), ribosome protein (4/148 = 2.7%), and there were also a variety of genes with other known or unknown functions in the biological system. For each gene listed in table S1, the systematic name, common name of the gene from *S. pombe* (if available), along with a brief description of the function of each gene product were also indicated. For convenience, we named the genes after their *S. cerevisiae* counterparts as the common name of the gene from *S. pombe* is not available.

### VPA and SB Increased Histone Acetylation and Atf1 Transcriptional Activity in a Clr6-dependent Manner

It is well established that HDAC inhibitor induces two important changes within the cell (i) an increase in the amount of hyperacetylated histones [32] and (ii) an increase in the level of transcription of certain genes [8]. As in mammalian cells both VPA and SB are classified as HDAC inhibitors, and in fission yeast overexpression of the *clr6^+^* gene, encoding class I HDAC caused a reduction in the level of H4-acetylation [33], we compared the level of histone acetylation in wild-type cells and *clr6-1* mutants. We expected that *clr6-1* mutation leads to an increase in histone acetylation because a defect in deacetylation. As expected, histone H4 acetylation was significantly increased in *clr6-1* mutants compared with wild-type cells (Figure 2B), indicating that the *clr6-1* mutation induces histone hyperacetylation. We expected VPA or SB treatment would increase histone acetylation in wild-type cells if they function as HADC inhibitor in *S. pombe*. We examined the levels of histone H4 acetylation (Lys5, Lys8 or Lys12) in wild-type cells treated with VPA or with SB, using TSA as a control. Similar to TSA treatment, both VPA and SB treatment resulted in a significant increase in the levels of acetylation at the three lysine residues tested on H4 tails compared with the wild-type control (Figure 2C), suggesting that both of the two drugs inhibit HDAC in fission yeast. Notably, TSA treatment significantly increased histone H4 acetylation compared with VPA or SB (Figure 2C), indicating that TSA is a more potent HDAC inhibitor. Among the total 148 VPA-sensitive mutants, only 38 deletion strains displayed varying levels of sensitivities to TSA. We hypothesize that TSA specifically inhibits HDAC whereas VPA affects a variety of biological processes besides HDAC activity.

**Figure 3:**
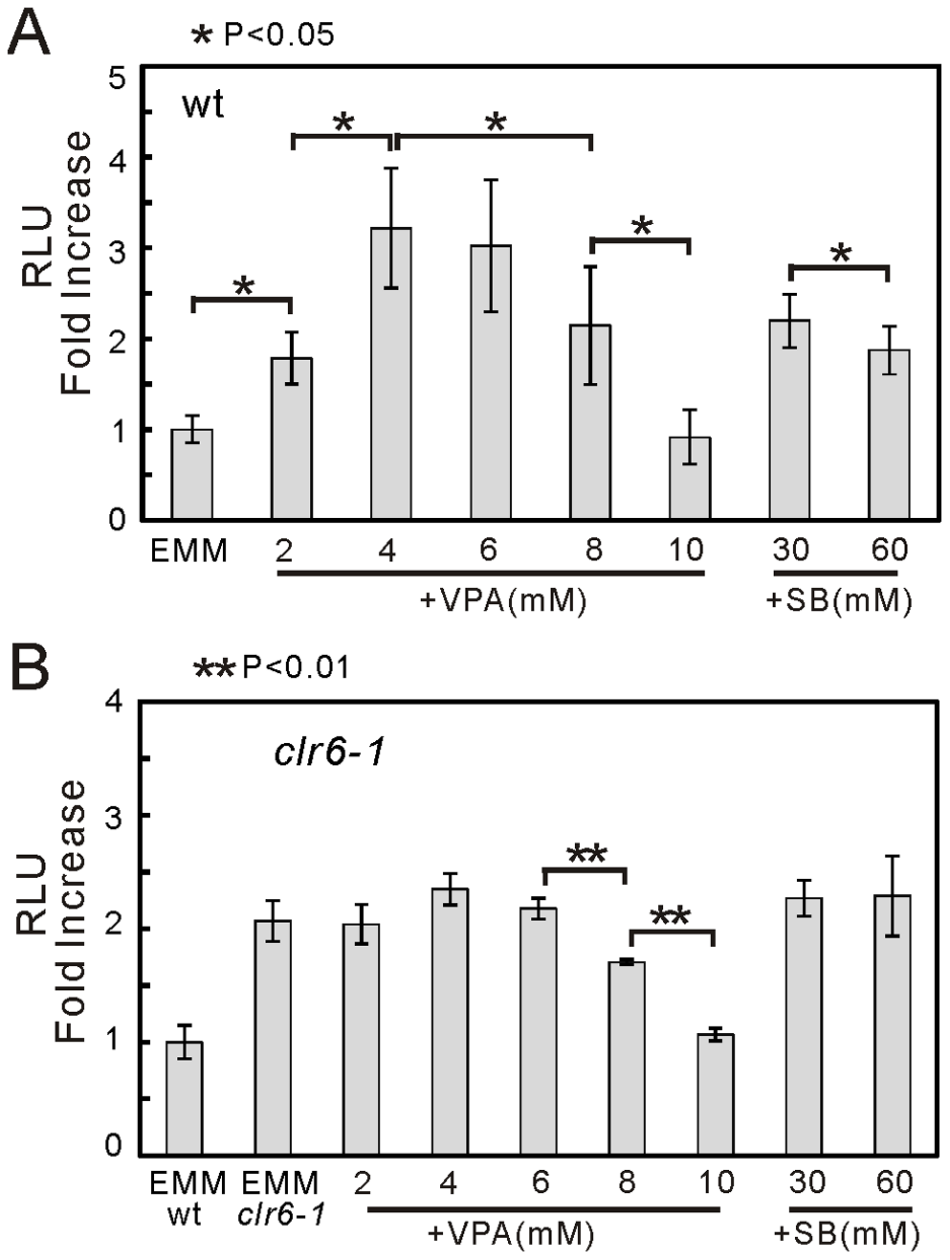
VPA and SB increased Atf1 transcriptional activity in a Clr6-dependent manner. (A) VPA or SB treatment markedly increased Atf1 transcription activity in wild-type cells. The wild-type cells harboring the multicopy plasmid (3XCRE::luc(R2.2) report plasmid) were grown to exponential phase, and assayed as described in Materials and Methods. The luminescence was followed for 5 hours. The data represent the accumulated value ratio of each sample (VPA or SB treatment) to the basal (EMM). Standard deviations are from three independent experiments, and each sample was analyzed in triplicate. Mean±S.D. (n = 6). P<0.05. (B) VPA or SB treatment failed to increase Atf1 transcription activity in *clr6-1* mutant cells. The experiments were performed as described in Figure 3A, except *clr6-1* mutant cells were monitored instead of the wild-type cells. Mean±S.D. (n = 6). P<0.01.

We previously monitored Atf1 transcription activity in living fission yeast cells using luciferase reporter genes [20]. It prompted us to monitor whether VPA and SB would increase the transcriptional activity of Atf1 in wild-type cells. Results showed that the Atf1 activity is markedly induced by VPA or SB treatment (Figure 3A). Notably, 4 mM VPA treatment induced a higher Atf1 activity than that of 8 mM VPA (P<0.05), and 30 mM SB treatment induced a higher Atf1 activity than that of 60 mM SB (P<0.05). We conjecture that at high concentration, VPA and SB are likely to target much broader targets such as nonhistone proteins that may lead to inhibit Atf1 transcription.

Next we monitored the transcriptional activity of Atf1 in *clr6-1* mutants. The result showed that VPA- and SB-induced Atf1 activation was hardly observed in *clr6-1* mutants (Figure 3B). Notably, the basal of Atf1 transcription activity in *clr6-1* mutants is about 2-fold of that in wild-type cells (Figure 3B). We hypothesize that *clr6-1* mutants already have elevated levels of Atf1 activity so cells are “preconditioned” and VPA addition does not impose the analogous responses as that observed in wild-type cells. Additionally, increased concentration of VPA resulted in the same trend of Atf1 transcriptional activity (Figure 3B, P<0.01) as observed in wild-type cells (Figure 3A). These results indicate that VPA and SB increased histone acetylation and Atf1 transcriptional activity in a Clr6-dependent manner.

### VPA, but not SB, Caused an Increase in the Cytoplasmic Ca2+ Level Due to Ca2+ Influx from the Extracellular Medium

**Figure 4:**
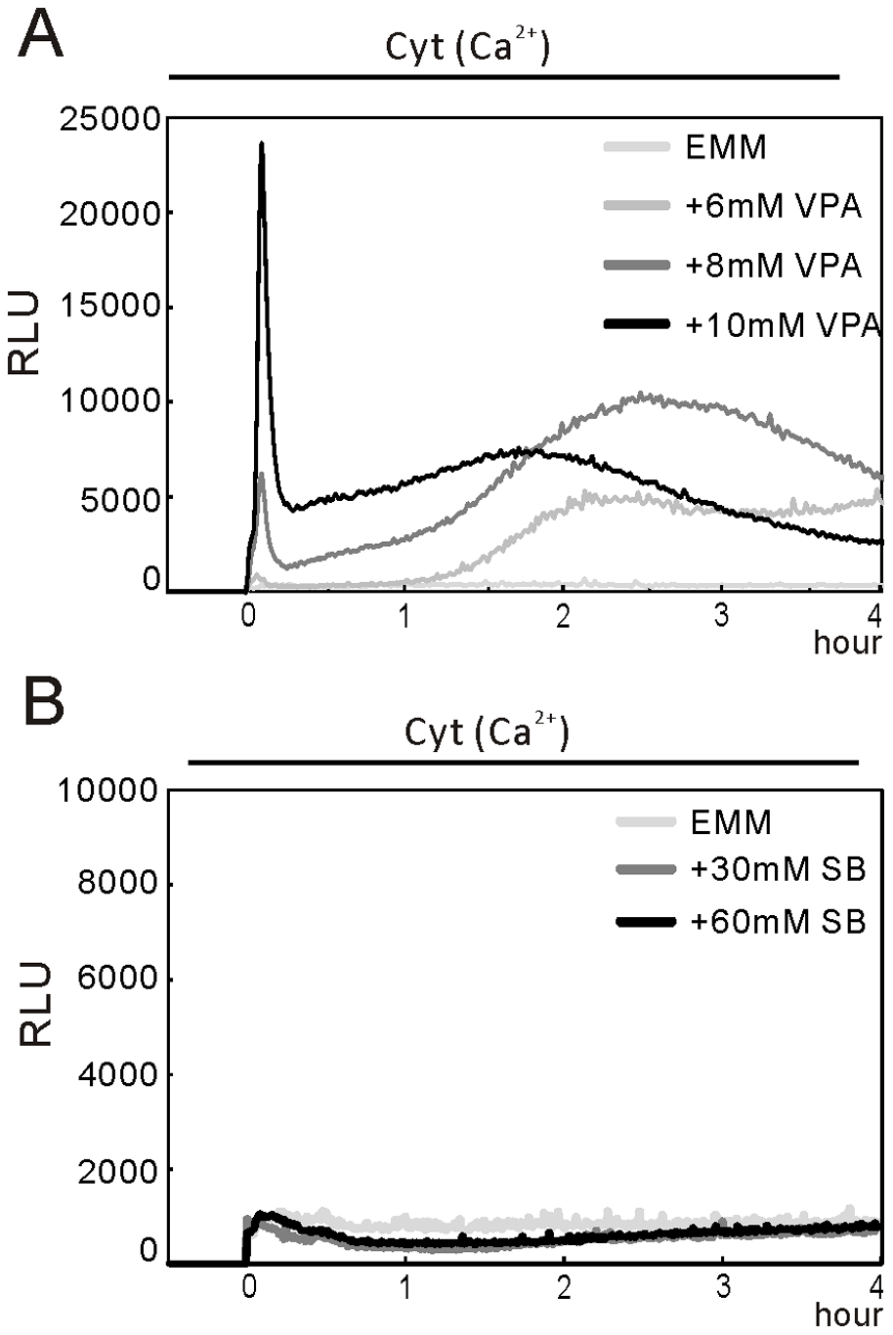
VPA, but not SB, caused an increase in the cytoplasmic Ca^2+^ level. (A) VPA caused an increase in the cytoplasmic Ca^2+^ level. The wild-type cells harboring *adh1*-GFP-19-AEQ (pKB6892) were grown to exponential phase, and then the cells were collected and treated as described in Materials and Methods. A 10µl volume of EMM or 10X stock of various concentration of VPA (A) or SB (B) was added into the 96-well plate, and the cells were delivered to the wells via the luminometer pump. The aequorin luminescence was followed for 4 hours. The luminescence, given as relative light units (RLU) s^−1^, is plotted versus time. The data are representative of six independent experiments. (B) SB didn’t cause an increase in the cytoplasmic Ca^2+^ level. The experiments were performed as described in Figure 4A except that SB was used as stimulant instead of VPA. The data are representative of six independent experiments.

We have previously demonstrated the Ca^2+^ influx activated calcineurin and caused nuclear translocation of transcription factor Prz1 [34]. We also found that VPA treatment had a similar effect on Prz1 localization [13]. Recently, we monitored the cytoplasmic Ca^2+^ levels in living fission yeast cells by a high-sensitivity assay [21]. These prompted us to monitor the cytoplasmic Ca^2+^ level in wild-type cells treated with VPA or SB. Results showed that VPA induced a dose-dependent increase in the cytoplasmic Ca^2+^ level (Figure 4A) whereas SB failed to induce cytoplasmic Ca^2+^ increase (Figure 4B). VPA induced a two-phase increase in cytoplasmic Ca^2+^ level. The first phase is an acute dose-dependent burst peaking within 5 minutes with a subsequent rapid decline and the second phase is a less pronounced peaking at 2–3 hour with a slow increase and a subsequent slow decline. The treatment of VPA, but not SB, induced cytoplasmic Ca^2+^ increase, suggesting that VPA may regulate the cytoplasmic Ca^2+^ level independent on its HDAC inhibition.

To investigate whether VPA-induced Atf1 activity is related to cytoplasmic Ca^2+^ increase, we examined the effect of rapid Ca^2+^ chelator BAPTA (1, 2-bis (o-aminophenoxy) ethane-N, N, N’, N’-tetraacetic acid) on VPA-induced Atf1 activity in wild-type cells. Results showed that VPA-induced Atf1 activity was inhibited by the addition of BAPTA in a dose-dependent manner (Figure S2), indicating that the increase in Atf1 activity and cytoplasmic Ca^2+^ level upon VPA treatment are related. We next monitored the cytoplasmic Ca^2+^ level in Δ*atf1* cells upon VPA treatment. Results showed that VPA induced a dose-dependent increase in the cytoplasmic Ca^2+^ level similar to that observed in wild-type cells (Figure S3 and S5), indicating that Atf1 activity has no significant effect on cytoplasmic Ca^2+^ level.

We previously demonstrated that VPA treatment resulted in cell-wall damage [13]. Cell-wall damage triggers a Ca^2+^ influx through the Cch1-Yam8 channel complex [21]. In present study, we demonstrated that VPA induces the increase of cytoplasmic Ca^2+^ level (Figure 4A), and VPA-induced Atf1 activity was inhibited by the addition of BAPTA (Figure S2). These data indicated that VPA-induced Atf1 activity could be related to cell-wall damage induced by VPA.

In the present screen, 55 strains showed sensitivity to VPA, but not SB. Also we show that VPA, but not SB, induced the Ca^2+^ cytoplasmic influx. It prompted us to investigate whether the calcium sensitivity of 55 mutants is linked to this effect. We compared the growth of wild-type cells with 148 VPA- or SB-sensitive strains on YPDA containing 0.2 mM CaCl_2_ plates. Among 55 VPA-sensitive mutants, 9 mutants showed varying levels of CaCl_2_ sensitivity, and among 93 VPA- and SB-sensitive mutants, 38 mutants showed varying levels of CaCl_2_ sensitivity (Table S2).

**Figure 5:**
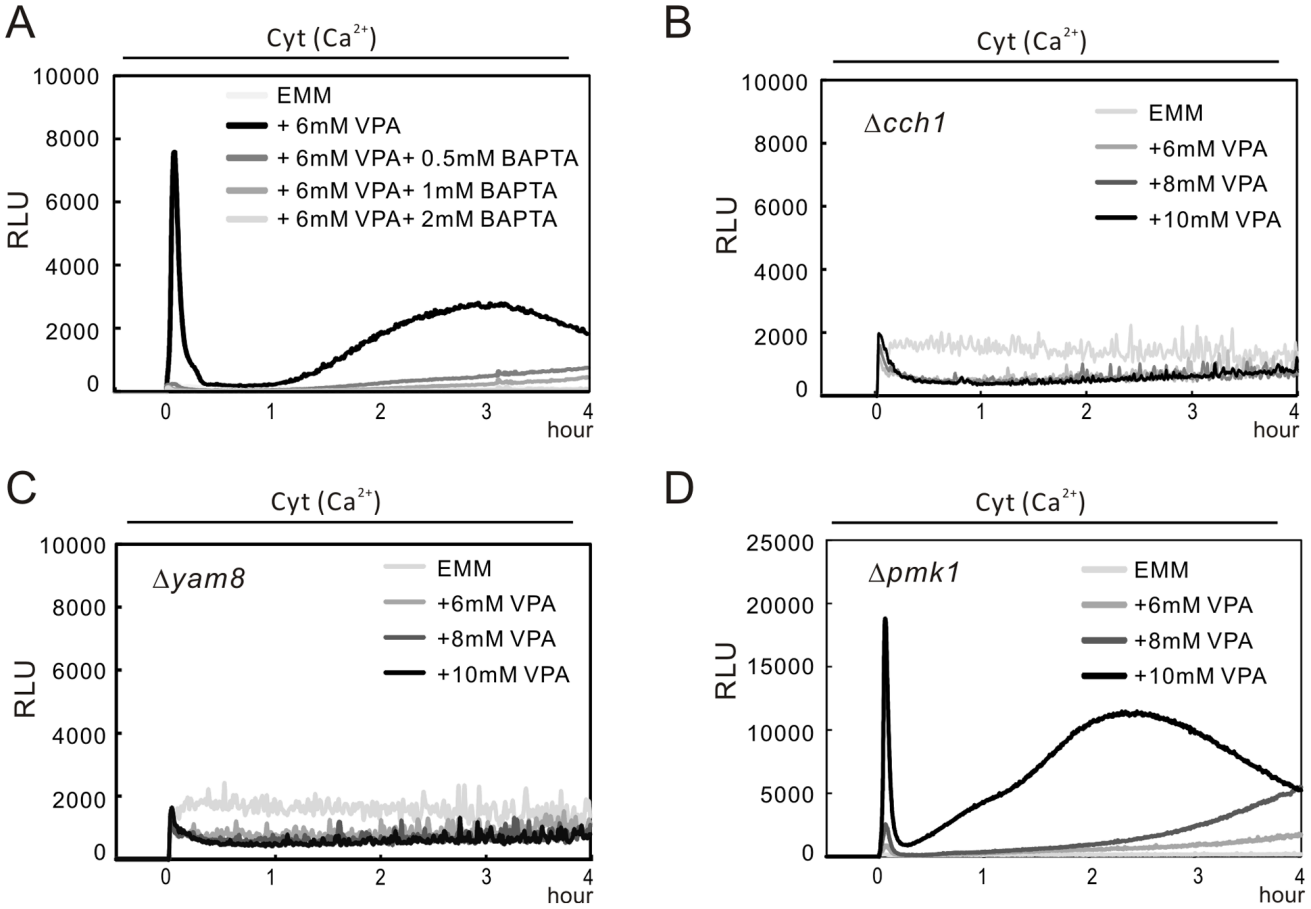
VPA triggers Ca^2+^ influx via the Cch1-Yam8 channel complex. (A) The increase in the cytoplasmic Ca^2+^ level is derived from the extracellular medium. The experiment was performed as described in Figure 4, expect that prior to the addition of 6 mM VPA, various concentrations of BAPTA (0.5, 1 and 2 mM) were added to chelate Ca^2+^ in EMM medium. The data are representative of six independent experiments. (B) The *cch1* deletion abolished VPA-induced Ca^2+^ influx. The Δ*cch1* cells harboring pKB6892 were cultured and assayed as described in the legend of Figure 4A. The data are representative of six independent experiments. (C) The *yam8* deletion abolished VPA-induced Ca^2+^ influx. The Δ*yam8* cells harboring pKB6892 were cultured and assayed as described in the legend of Figure 4A. The data are representative of six independent experiments. (D) The VPA-induced Ca^2+^ influx was observed in Δ*pmk1*. The Δ*pmk1* cells harboring pKB6892 were cultured and assayed as described in Figure 4A. The data are representative of six independent experiments.

To investigate whether the increase in cytoplasmic Ca^2+^ level is due to the influx from extracellular medium or due to the release from an internal store, we examined the effect of BAPTA by the same assay. Our result showed that the increase was inhibited by the addition of BAPTA in a dose-dependent manner (Figure 5A), indicating that the increase in the cytoplasmic Ca^2+^ level is dependent on the influx across the channel that exists on the plasma membrane.

### VPA Increased the Cytoplasmic Ca2+ Level via Cch1- Yam8 Channel Complex

We have previously demonstrated that the deletion of ***cch1*** ^+^ gene that encodes putative subunit of a Ca^2+^ channel, abolished the NaCl-induced activation of calcineurin and the synergistic increase caused by NaCl plus FK506 via affecting Ca^2+^ influx [35] [21]. It prompted us to investigate whether VPA-induced cytoplasmic Ca^2+^ increase is mediated by the Cch1-Yam8 channel complex. In Δ*cch1* cells, VPA failed to induce the increase in the cytoplasmic Ca^2+^ level (Figure 5B), which is in accord with the results in Δ*yam8* cells (Figure 5C). Our data suggested that VPA affects intracellular calcium concentration via Cch1-Yam8 channel complex. Consistently, it is demonstrated that in hippocampal slices, VPA has an effect on the entry of Ca^2+^ into nerve endings [36].

We previously demonstrated that Pmk1 MAPK positively regulates the Cch1-Yam8 channel complex upon NaCl treatment [21]. It prompted us to examine the cytoplasmic Ca^2+^ level in Δ*pmk1* cells. The results showed that VPA-induced Ca^2+^ increase was also observed in Δ*pmk1* cells (Figure 5D and S5). We also examine the effect of VPA on the cytoplasmic Ca^2+^ level in Δ*mkh1* and Δ*pck2* cells that do not show VPA sensitivities. Results showed that in Δ*mkh1* and Δ*pck2* cells, VPA induced a dose-dependent increase in the cytoplasmic Ca^2+^ level similar to that observed in wild-type cells (Figure S1A and S1B). As Cch1-Yam8 channel complex is dephosphorylated by calcineurin [23], it prompted us to investigate whether VPA affect Ca^2+^ influx in the knockout cells of the *ppb1* ^+^ gene, encoding a single catalytic subunit of fission yeast calcineurin. Results showed that in Δ*ppb1* cells, VPA also induced a dose-dependent increase in the cytoplasmic Ca^2+^ level (Figure S1C and S5). It should be noted that in Δ*ppb1* cells, the relative light units (RLU) of basal and peak were significantly higher than that in the wild-type cells (Figure S1C and S5, EMM). Our present results suggest that VPA treatment induces the cytoplasmic Ca^2+^ increase via Cch1-Yam8 channel complex. Previously, we demonstrated that VPA affects membrane trafficking, which leads to the enhanced sensitivity to cell-wall damage [13]. We hypothesize that VPA induces the increase of cytoplasmic Ca^2+^ level in addition to cell-wall damage.

### VPA-sensitive Mutants Involved in Membrane Trafficking

Our previous screen for VPA-sensitive mutants resulted in the isolation of 3 membrane trafficking defective mutants, namely *vas1/vps45* [13], *vas2/aps1* [14] and *vas3/ric1* [15]. In the present screen, 11.5% (17/148 genes) of the VPA-sensitive mutants showed defects in intracellular membrane trafficking (Table S1). According to the *S. pombe* GeneDB (http://old.genedb.org/genedb/pombe/), most of the genes are involved in Golgi/endosome membrane trafficking. Firstly, four genes encode adaptins, specifically, two genes encode AP-1 (adaptor protein complex-1) subunits (Apm1 and Apl4), and the other two genes encode AP-3 subunits (Aps3 and Apl6). Interestingly, these four strains also showed sensitivities to SB (Table S1). Previously we demonstrated that VPA affects membrane trafficking which leads to the enhanced sensitivity to cell-wall damage in fission yeast [13]. We also examined whether SB treatment could enhance the sensitivity to cell-wall damage. The result showed that SB slightly affected cell-wall integrity (Figure S4). We hypothesize that VPA and SB primarily affect cell-wall integrity and thereby cause sensitivities in various mutant backgrounds. Additionally, they enter the cells and thereby affect membrane trafficking through HDAC inhibition. Secondly, four genes encode the subunits of retromer complex (Vps5, Vps26p, Vps29p and Vps35). In budding yeast, the retromer complex consisting of 5 subunits (Vps5p, Vps17p, Vps26p, Vps29p and Vps35p) is responsible for the retrograde transport of vacuolar protein sorting receptor Vps10 from endosomes to the TGN [37], [38]. The subcomplex containing Vps26p, Vps29p and Vps35p is required for cargo recognition/selection and the other subcomplex containing Vps5p and Vps17p is required for membrane deformation [39], [40]. Up to date, the retromer complex is involved in the recycling of Fet3p (*S. cerevisiae*), Ftr1p (*S. cerevisiae*), Can1p (*S. cerevisiae*), Vps10 (*S. cerevisiae* and *S. pombe*) and the sorting receptor Wntless (*C. elegans*). In fission yeast deletion of the subunits of retromer complex (Vps26, Vps29 and Vps35) subunit confer the sensitivity to thiabentazole [41]. In budding yeast, deletion of the subunits of retromer complex showed the resistance to canavanine, a toxic analog of arginine [40]. Here we showed deletion of the subunits of retromer complex showed sensitivities to VPA, but not SB, suggesting VPA may affect retromer complex independent of HDAC inhibition. An alternative possibility is that VPA and SB have differential effect or differential targeting of HDACs. Consistently, VPA results in proteasomal degradation of HDAC2 [42] while SB results in the degradation of HDAC4 [43].

### VPA-sensitive Mutants Involved in Signaling Transduction

In the present screen, 12.8% (19/148) of the genes associated with increased sensitivity to VPA were shown to be involved in signaling transduction pathways (Table S1). Two genes encode small GTPase Ras1 and Rho3, and Rho GDP dissociation inhibitor Rdi1 and Rho3 interaction partner formin For3 were also isolated. Interestingly, Δ*rho3* cells showed sensitivities to both VPA and SB, whereas Δ*rdi1* and Δ*for3* cells only show hypersensitivity to VPA. Consistently, Kita et al. demonstrated that Rho3 is involved in Golgi/Endosome trafficking through functional interaction with adaptin [44] and Nakano et al. demonstrated that Rho3 and formin For3 function in polarized cell growth [45]. Rho3 is involved in membrane trafficking, actin cytoskeleton and cytoplasmic microtubules, whereas For3 is involved in actin cytoskeleton and cytoplasmic microtubules. Another two important genes encode the members of Pmk1 MAPK cell-wall integrity. Pek1 encodes MAP kinase kinase and Pmk1 encodes MAP kinase. To our surprise Δ*pek1* and Δ*pmk1* are severely sensitive (+++) to both VPA and SB whereas deletion of the *mkh1^+^*gene encoding MAPK kinase kinase, *pck2* ^+^ gene encoding protein kinase C, and the *rho2^+^*gene encoding small GTPase, didn’t show sensitivity to neither VPA nor SB. Further studies are needed to uncover the mechanisms why MAPKK and MAPK deletion, but not MAKKK deletion, show sensitivities to VPA and SB.

## Supporting Information

Figure S1 **VPA caused an increase in the cytoplasmic Ca^2+^ level in Δ** ***mkh1*** **, Δ** ***pck2*** **and Δ** ***ppb1*** **cells.** The Δ*mkh1* (A), Δ*pck2* (B) or Δ*ppb1* cells (C) harboring pKB6892 were cultured and assayed as described in Figure 4A. The data are representative of three independent experiments.
(TIF)

Figure S2 **VPA-induced Atf1 activity was inhibited by BAPTA.** The experiment was performed as described in Figure 3, expect that prior to the addition of 4 mM VPA, various concentrations of BAPTA (0.5, 1 and 2 mM) were added to chelate Ca^2+^ in EMM medium. The data are representative of three independent experiments. Standard deviations are from three independent experiments.
(TIF)

Figure S3 **VPA caused an increase in the cytoplasmic Ca2+ level in Δatf1 cells.** The Δ*atf1* cells harboring pKB6892 were cultured and assayed as described in Figure 4A. The data are representative of three independent experiments.
(TIF)

Figure S4 **Effect of SB on cell-wall digestion by β-glucanase.** Wild-type cells were assayed for zymolyase sensitivity with or without SB treatment. The Δpmk1 cells were assayed as a positive control. The wild-type cells and Δpmk1 cells were cultured in YPD at 27°C for 10 hours to exponential phase. Then the wild-type and Δpmk1cells were treated with the indicated concentrations of SB for 6 hours and incubated with 100 µg/ml of β-glucanase (zymolyase 20T) at 27°C with vigorous shaking. Cell lysis was monitored by measuring optical density at 660 nm. The value before adding the enzyme was taken as 100%. The data are representative of three independent experiments.
(TIF)

Figure S5 **VPA induced an increase in the cytoplasmic Ca2+ level in wild-type, Δpmk1, Δatf1 and Δppb1 cells.** The wild-type, Δpmk1, Δatf1 and Δppb1 cells harboring pKB6892 were cultured and assayed as described in Figure 4A. The data represent the accumulated value ratio of each sample treated with the indicated concentrations of VPA to the basal (EMM) of wild-type cells. Standard deviations are from three independent experiments.
(TIF)

Table S1 **Summary of the gene name and products of VPA- and/or SB-sensitive mutants.**
(DOCX)

Table S2 **S. pombe genes identified in the screen for CaCl2-sensitivity in 148 sensitive strains.**
(DOCX)
