# Supplementary figures and images for: Genome-Wide Screening for Genes Associated with Valproic Acid Sensitivity in Fission Yeast
Source: PLoS One. 2013 Jul 5;8(7):e68738. doi: 10.1371/journal.pone.0068738 (PMC3702616; doi:10.1371/journal.pone.0068738)

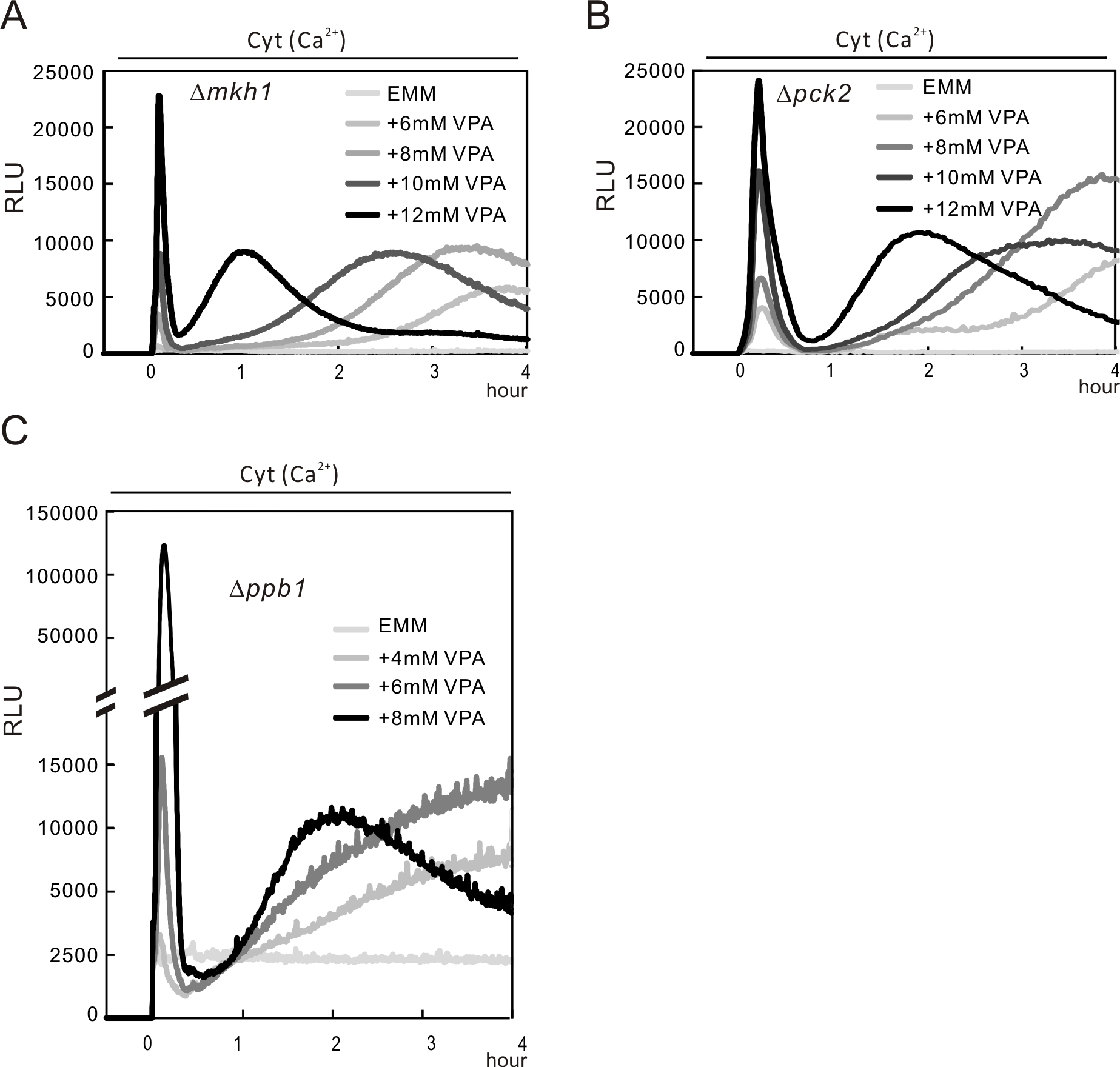

Supplement: Figure S1 — VPA caused an increase in the cytoplasmic Ca2+ level in Δ mkh1 , Δ pck2 and Δ ppb1 cells. The Δmkh1 (A), Δpck2 (B) or Δppb1 cells (C) harboring pKB6892 were cultured and assayed as described in Figure 4A. The data are representative of three independent experiments. (TIF) [file pone.0068738.s001.tif]

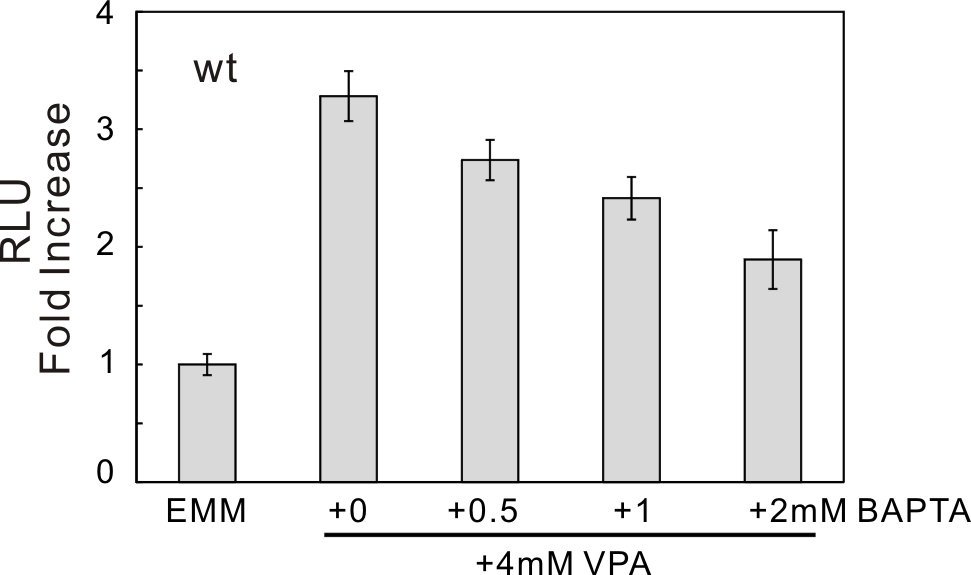

Supplement: Figure S2 — VPA-induced Atf1 activity was inhibited by BAPTA. The experiment was performed as described in Figure 3, expect that prior to the addition of 4 mM VPA, various concentrations of BAPTA (0.5, 1 and 2 mM) were added to chelate Ca2+ in EMM medium. The data are representative of three independent experiments. Standard deviations are from three independent experiments. (TIF) [file pone.0068738.s002.tif]

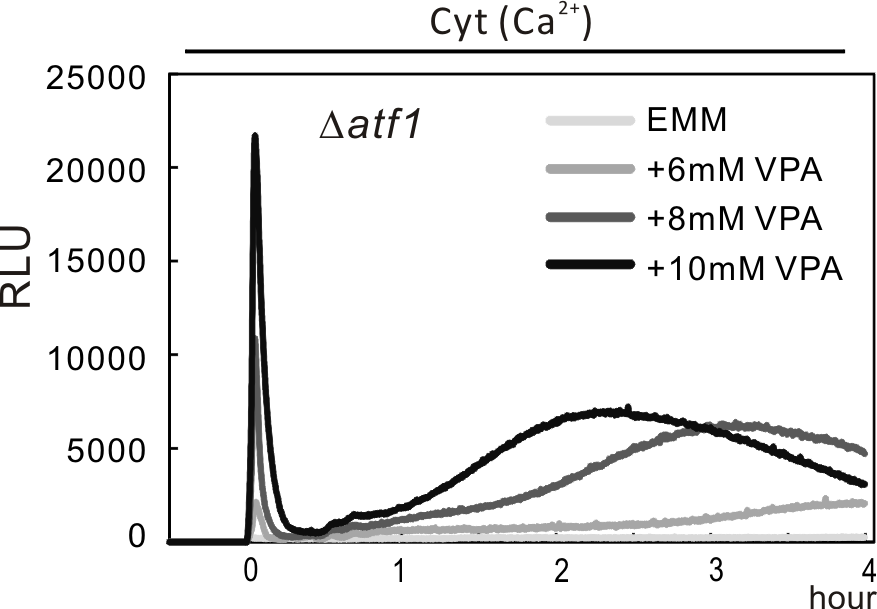

Supplement: Figure S3 — VPA caused an increase in the cytoplasmic Ca2+ level in Δatf1 cells. The Δatf1 cells harboring pKB6892 were cultured and assayed as described in Figure 4A. The data are representative of three independent experiments. (TIF) [file pone.0068738.s003.tif]

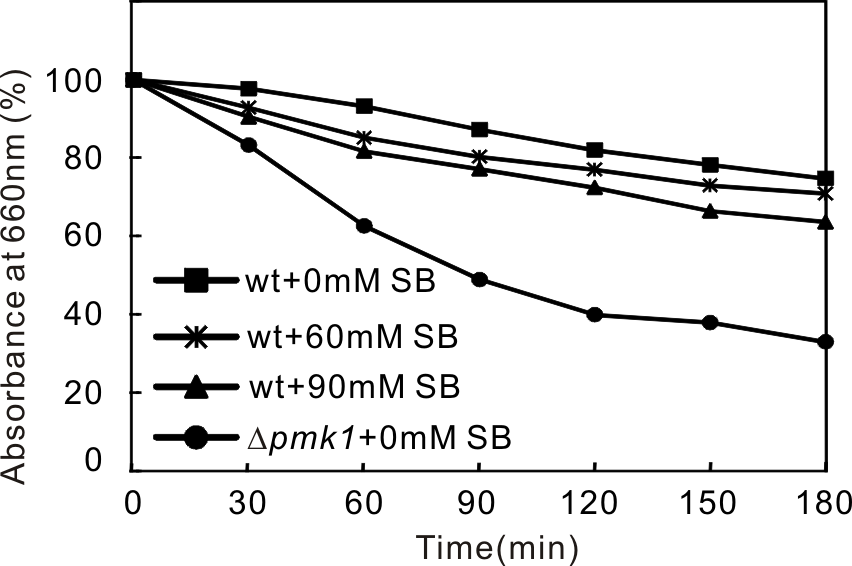

Supplement: Figure S4 — Effect of SB on cell-wall digestion by β-glucanase. Wild-type cells were assayed for zymolyase sensitivity with or without SB treatment. The Δpmk1 cells were assayed as a positive control. The wild-type cells and Δpmk1 cells were cultured in YPD at 27°C for 10 hours to exponential phase. Then the wild-type and Δpmk1cells were treated with the indicated concentrations of SB for 6 hours and incubated with 100 µg/ml of β-glucanase (zymolyase 20T) at 27°C with vigorous shaking. Cell lysis was monitored by measuring optical density at 660 nm. The value before adding the enzyme was taken as 100%. The data are representative of three independent experiments. (TIF) [file pone.0068738.s004.tif]

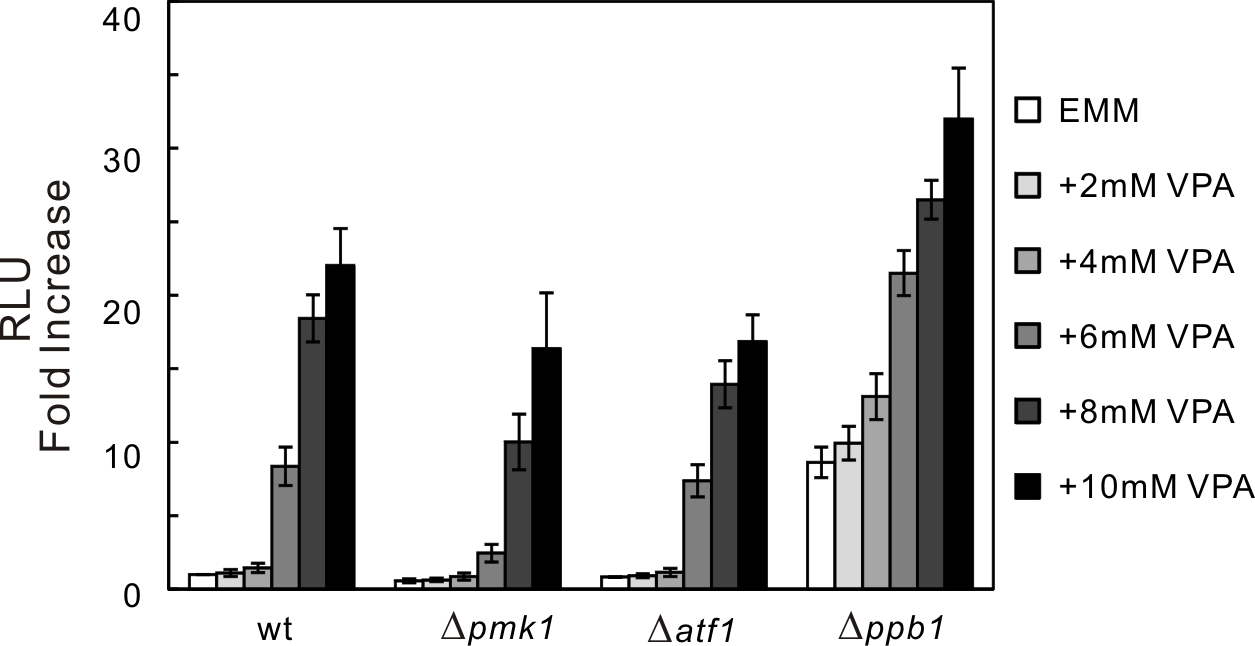

Supplement: Figure S5 — VPA induced an increase in the cytoplasmic Ca2+ level in wild-type, Δpmk1, Δatf1 and Δppb1 cells. The wild-type, Δpmk1, Δatf1 and Δppb1 cells harboring pKB6892 were cultured and assayed as described in Figure 4A. The data represent the accumulated value ratio of each sample treated with the indicated concentrations of VPA to the basal (EMM) of wild-type cells. Standard deviations are from three independent experiments. (TIF) [file pone.0068738.s005.tif]
