# Supplementary material for: Genome-Wide Screening for Genes Associated with Valproic Acid Sensitivity in Fission Yeast
Source: PLoS One. 2013 Jul 5;8(7):e68738. doi: 10.1371/journal.pone.0068738 (PMC3702616; doi:10.1371/journal.pone.0068738)
Supplement: Table S1 — Summary of the gene name and products of VPA- and/or SB-sensitive mutants. (DOCX) [file pone.0068738.s006.docx]

**Table S1 Summary of the gene name and products of VPA- and/or SB-sensitive mutants**

| **Function category** | | | | | |
| --- | --- | --- | --- | --- | --- |
| **VPA sensitivity** | **SB sensitivity** | **TSA**  **sensitivity** | **Systematic Name** | **Gene Name** | **Product** |
| DNA and RNA metabolism | | | | | |
| **+++** | **+++** | ++ | SPCC24B10.11c | mft1 | THO complex subunit 7 |
| **+++** | **+++** | + | SPCC757.09c | rnc1 | RNA-binding protein that suppresses calcineurin deletion Rnc1 |
| **+++** | **+++** |  | SPBC13E7.08c | leo1^☆^ | RNA polymerase II associated Paf1 complex |
| **+++** | **++** | + | SPCC31H12.08c* | ccr4* | CCR4-Not complex subunit Ccr4 |
| **++** | **++** | + | SPCC11E10.06c | elp4 | RNA polymerase II elongator complex subunit Elp4 |
| **++** | **++** |  | SPAC20H4.03c | tfs1 | transcription elongation factor TFIIS |
| **++** | + | ++ | SPAC1006.03c | red1 | RNA elimination defective protein Red1/human CCDC131 homolog |
| **+** | **++** | +++ | SPAC29B12.06c | rcd1 | RNA-binding protein Rcd1 |
| **+** | **++** |  | SPCC74.09 | mug24 | RNA-binding protein |
| **+** | **++** |  | SPCC74.02c | **N/A** | mRNA cleavage and polyadenylation specificity factor complex associated protein |
| **+** | **++** |  | SPCC736.07c* | bud27^☆^* | unconventional prefoldin involved in translation initiation |
| **+** | **++** | + | SPAPB1E7.02c | mcl1 | DNA polymerase alpha accessory factor Mcl1 |
| **+** | + |  | SPAC19G12.02c | pms1 | MutL family mismatch-repair protein Pms1 |
| **+** | + |  | SPBC1778.02 | rap1 | telomere binding protein Rap1 |
| **+** |  |  | SPBC30B4.06c | mto1^☆^ | mitochondrial GIDA family tRNA uridine 5-carboxymethylaminomethyl modification enzyme |
| **+** |  | + | SPBC56F2.08c | **N/A** | RNA-binding protein |
| **+** |  |  | SPAC140.02 | gar2 | nucleolar protein required for rRNA processing |
| **+** |  |  | SPBC16E9.12c | pab2 | poly(A) binding protein Pab2 |
| **+** |  |  | SPBC16G5.02c | **N/A** | ribokinase |
| **+** |  |  | SPAC2C4.07c | **N/A** | ribonuclease II (RNB) family |
| **+** |  |  | SPBC337.03 | rtt103^☆^ | RNA polymerase II transcription termination factor |
| **+** |  |  | SPAC6G9.14 | **N/A** | RNA-binding protein |
| **+** |  |  | SPAPYUG7.04c | rpb9 | DNA-directed RNA polymerase II complex subunit Rpb9 |
| **+** |  |  | SPBC2D10.16 | mhf1 | FANCM-MHF complex subunit Mhf1 |
| **Signal transduction** | | | | | |
| +++ | +++ | + | SPBC543.07 | pek1 | MAP kinase kinase Pek1 |
| +++ | +++ |  | SPBC119.08 | pmk1 | MAP kinase Pmk1 |
| +++ | +++ |  | SPBC725.09c | hob3 | BAR adaptor protein Hob3 |
| +++ |  |  | SPAC6F12.06 | rdi1 | Rho GDP dissociation inhibitor Rdi1 |
| ++ | ++ |  | SPBC12C2.02c | ste20 | Rictor homolog, Ste20 |
| ++ | ++ |  | SPBC1D7.03 | mug80 | cyclin Clg1 |
| ++ | + |  | SPCC777.08c | bit61 | TORC2 subunit Bit61 |
| + | +++ |  | SPAC630.13c | tsc2 | tuberin |
| + | ++ | +++ | SPBC4F6.06 | kin1 | microtubule affinity-regulating kinase Kin1 |
| + | ++ | + | SPAC22F3.13 | tsc1 | hamartin |
| + | ++ |  | SPAC17H9.09c | ras1 | GTPase Ras1 |
| + | ++ |  | SPAC8E11.02c | rad24 | 14-3-3 protein Rad24 |
| + | + |  | SPAC23C4.08 | rho3 | Rho family GTPase Rho3 |
| + |  |  | SPAC23H4.17c | srb10 | cyclin-dependent protein kinase Srb10 |
| + |  |  | SPCC297.05 | **N/A** | diacylglycerol binding protein |
| + |  |  | SPBC11B10.07c | ivn1 | CDC50 domain protein, implicated in signal transduction |
| + |  | ++ | SPCC895.05 | for3 | formin For3 |
| + |  | + | SPBC4F6.12 | pxl1 | paxillin-like protein Pxl1 |
| + |  |  | SPBC16D10.08c | **N/A** | heat shock protein Hsp104 |
| **Membrane trafficking** | | | | | |
| +++ | ++ | + | SPBC530.01 | gyp1 | GTPase activating protein Gyp1 |
| +++ | + | ++ | SPBP16F5.07 | apm1 | AP-1 adaptor complex subunit Apm1 |
| +++ | + | + | SPCPJ732.01 | vps5 | retromer complex subunit Vps5 |
| +++ |  | ++ | SPBC23E6.08 | sat1 | Golgi membrane exchange factor subunit Sat1 |
| +++ |  |  | SPCP1E11.06 | apl4 | AP-1 adaptor complex gamma subunit Apl4 |
| ++ | + |  | SPAC2G11.03c | vps45 | vacuolar sorting protein Vps 45 |
| ++ | + |  | SPAC9E9.14 | vps24 | vacuolar sorting protein Vps24 |
| ++ |  | +++ | SPAC15E1.06 | vps29 | retromer complex subunit Vps29 |
| + | +++ |  | SPAC30D11.05 | aps3 | AP-3 adaptor complex subunit Aps3 |
| + | + |  | SPAC23H3.06 | apl6 | AP-3 adaptor complex subunit Apl6 |
| + | + |  | SPBC1105.08 | emp70 | EMP70 family (Endomembrane protein 70 ) |
| + | + |  | SPAC1527.02 | sft2 | Golgi transport protein Sft2 |
| + |  | +++ | SPAC4G9.13c | vps26 | retromer complex subunit Vps26 |
| + |  | +++ | SPBC4F6.10 | vps901 | guanyl-nucleotide exchange factor Vps901 |
| + |  | + | SPBC119.12 | rud3 | Golgi matrix protein Rud3 |
| + |  |  | SPCC126.08c | **N/A** | lectin family glycoprotein receptor |
| + |  |  | SPAC1F5.05c | mso1 | secretory vesicle docking component Mso1 |
| **Chromatin remodeling** | | | | | |
| +++ | + |  | SPBC13G1.08c | ash2 | Ash2-trithorax family protein |
| +++ |  |  | SPCC594.05c | Spf1 | COMPASS complex subunit/Set1C PHD Finger protein Spf1 |
| ++ | ++ | +++ | SPBC11B10.10c | pht1 | histone H2A variant |
| ++ | + | ++ | SPAC22E12.11c | set3 | histone lysine methyltransferase Set3 |
| + | +++ |  | SPCC16C4.20c | nhp10 | Ino80 complex subunit |
| + | ++ | +++ | SPBC29A3.05 | vps71 | Swr1 complex subunit Vps71 |
| + | ++ |  | SPAC10F6.08c | nht1 | Ino80 complex HMG box protein Nht1 |
| + | + | + | SPBC16D10.07c | sir2 | Sir2 family histone deacetylase Sir2 |
| + |  |  | SPCC126.13c | **N/A** | histone deacetylase complex subunit, SAP128 family |
| + |  |  | SPAC144.02 | iec1 | Ino80 complex subunit Iec1 |
| + |  |  | SPAC1071.02 | mms19 | TFIIH regulator |
| + |  |  | SPBC21C3.02c | dep1 | Sds3-like family |
| + |  |  | SPBC21D10.10 | bdc1 | bromodomain protein |
| + |  |  | SPBC30B4.04c | sol1 | SWI/SNF complex subunit Sol1 |
| + | + |  | SPCC364.06 | nap1 | nucleosome assembly protein Nap1/histone chaperone Nap1 |
| Mitochondrial function | | | | | |
| +++ | +++ |  | SPAC23C11.10* | usb1* | mitochondrial respiratory chain complex III assembly Usb1 |
| +++ | +++ |  | SPAC1071.11 | **N/A** | NADH-dependent flavin oxidoreductase |
| +++ | +++ |  | SPBC29A3.10c | atp14 | F1-ATPase subunit H |
| +++ | ++ |  | SPBC1289.09 | tim21 | mitochondrial inner membrane presequence translocase complex subunit Tim21 |
| ++ |  |  | SPBC30D10.14 | aim2^☆^ | dienelactone hydrolase family |
| + | ++ |  | SPAC1687.12c | coq4 | ubiquinone biosynthesis protein Coq4 |
| + | ++ |  | SPBC2G5.06c | hmt2 | sulfide-quinone oxidoreductase |
| + | ++ |  | SPBC2F12.12c | **N/A** | human c19orf29 ortholog |
| + | + |  | SPBC16H5.06 | rip1 | ubiquinol-cytochrome-c reductase complex subunit 5 |
| + |  |  | SPAC4G8.08 | mrs3^☆^ | iron ion transporter |
| + | + |  | SPCC11E10.04 | ppr6 | mitochondrial PPR repreat protein Ppr6/ATPase expression protein homolog |
| Ubiquitination | | | | | |
| +++ | +++ |  | SPAC6B12.07c | **N/A** | ubiquitin-protein ligase E3 |
| ++ |  |  | SPAPB17E12.04c | csn2 | COP9/signalosome complex subunit Csn2 |
| + | ++ | ++ | SPAC31G5.18c | sde2 | ubiquitin family, human C1ORF55 related |
| + | ++ | + | SPAC1782.01 | ecm29 | proteasome component Ecm29 |
| + | + | ++ | SPBC6B1.06c | ubp14 | ubiquitin C-terminal hydrolase Ubp14 |
| + | + | ++ | SPAC12B10.03 | bun62 | WD repeat protein, human WDR20 family |
| + |  |  | SPBC354.10 | def1 | RNAPII degradation factor |
| + |  | + | SPAC15A10.11 | ubr11 | N-end-recognizing protein |
| + |  |  | SPBC215.03c | csn1 | COP9/signalosome complex subunit Csn1 |
| + |  |  | SPAC30D11.13 | hus5 | SUMO conjugating enzyme |
| Transcription | | | | | |
| +++ | +++ |  | SPBC1773.12 | **N/A** | transcription factor |
| +++ | ++ |  | SPAC630.14c | tup12 | transcriptional corepressor Tup12 |
| +++ | + |  | SPCC1393.08 | **N/A** | transcription factor( zf-GATA type ) |
| +++ |  | + | SPAC1783.07c | pap1 | transcription factor Pap1/Caf3 (bZIP family) |
| ++ | ++ | +++ | SPBC15D4.02 | **N/A** | transcription factor, zf-fungal binuclear cluster type |
| ++ | + |  | SPAC25B8.19c | **N/A** | transcription factor( zf-C2H2 type ) |
| ++ |  |  | SPAC18B11.10 | tup11 | transcriptional corepressor Tup11 |
| + |  |  | SPCC1739.01 | lee1 ^☆^ | zinc finger protein (zf-CCCH type) |
| Genes encoding transporters | | | | | |
| +++ | + |  | SPBC1271.10c | hol1^☆^ | membrane transporter |
| ++ | +++ |  | SPBC1683.03c | **N/A** | membrane transporter |
| + | ++ |  | SPBC609.04 | caf5 | spermine family transporter |
| + | + |  | SPAC5D6.09c | mug86 | acetate transporter |
| + |  |  | SPAC23C11.14 | zhf1 | zinc ion transporter Zhf1 |
| Ribosome protein | | | | | |
| ++ | + |  | SPAC1610.02c | **N/A** | mitochondrial ribosomal protein subunit L1 |
| + | + |  | SPAC959.08 | rpl2102 | 60S ribosomal protein L21 |
| + |  |  | SPAC25G10.06 | rps2801 | 40S ribosomal protein S28 |
| + |  |  | SPAC1952.02 | tma23 | ribosome biogenesis protein |
| **Variety of other known functions** | | | | | |
| +++ | +++ | ++ | SPBC3H7.12 | rav2 | RAVE complex subunit Rav2 |
| +++ | +++ |  | SPAC227.01c* | **N/A** | Erd1 homolog |
| +++ | ++ |  | SPAC19G12.08 | scs7 | fatty acid hydroxylase |
| +++ | ++ |  | SPAC9.02c | paa1^☆^ | N-acetyltransferase |
| +++ | ++ | + | SPBC29A10.16c | cyb5^☆^ | cytochrome b5 |
| +++ | + |  | SPBC3B8.03 | lys9^☆^ | saccharopine dehydrogenase |
| +++ |  |  | SPAC30C2.02 | mmd1 | deoxyhypusine hydroxylase |
| +++ |  |  | SPBC4C3.08 | mug136 | acetylglucosaminyltransferase |
| ++ | ++ |  | SPBC3E7.09 | slp1^☆^ | Sad1-UNC-like protein involved protein folding in the ER |
| ++ | ++ |  | SPBC4F6.11c | **N/A** | asparagine synthase |
| ++ | ++ |  | SPBC359.06 | mug14 | adducin |
| ++ | + |  | SPBC1604.08c | imp1 | importin alpha |
| + | +++ |  | SPCC622.12c | gdh1 | NADP-specific glutamate dehydrogenase |
| + | ++ |  | SPBP4H10.17c | mrps2 | carboxyl methyl esterase |
| + | ++ |  | SPBC3H7.03c | kgd1^☆^ | 2-oxoglutarate dehydrogenase (lipoamide) (e1 component of oxoglutarate dehydrogenase complex) |
| + | ++ |  | SPAC25B8.05 | deg1^☆^ | pseudouridylate synthase |
| + | + |  | SPBC354.07c | **N/A** | oxysterol binding protein |
| + | + |  | SPBC31F10.02 | **N/A** | thioesterase superfamily protein/acyl-CoA hydrolase |
| + | + |  | SPBC25B2.02c | mam1 | M-factor transporter Mam1 |
| + |  |  | SPBC106.17c | cys2 | homoserine O-acetyltransferase |
| + |  |  | SPCC794.03 | **N/A** | amino acid permease, unknown 13 |
| + |  |  | SPAC1805.16c | pnp1^☆^ | purine nucleoside phosphorylase |
| **Unknown functions** | | | | | |
| **+++** | **+++** | +++ | SPBPB2B2.14c | **N/A** | S. pombe specific DUF999 protein family 8 |
| **+++** | **+++** | ++ | SPAC19A8.11c | irc6 | recombination protein Irc6 |
| **+++** | **+** |  | SPAC3H5.08c | **N/A** | WD repeat protein Wdr44 family |
| **+++** |  |  | SPBC660.05 | wwm1^☆^ | conserved fungal protein |
| **++** | **+++** | ++ | SPBC1778.05c | **N/A** | sequence orphan |
| **++** |  |  | SPAC56F8.12 | **N/A** | conserved fungal protein |
| **+** | **++** |  | SPBC1198.03c | **N/A** | Golgin subfamily A member |
| **+** | **++** |  | SPAC1705.02 | **N/A** | human 4F5S homolog |
| **+** | **++** |  | SPAC18G6.13 | **N/A** | sequence orphan |
| **+** | **+** | + | SPAC6G9.15c | **N/A** | sequence orphan |
| **+** |  |  | SPAC8E11.05c | **N/A** | conserved fungal protein |
| **+** |  |  | SPAC29B12.11c | **N/A** | human WW domain binding protein-2 ortholog |
| **+** |  |  | SPAC6C3.07 | mug68 | sequence orphan |

**+++ indicates that the cells completely failed to grow on YPDA plates containing drugs plates.**

**++ indicates that tiny colonies were observed to grow on YPDA plates containing drugs plates.**

**+ indicates that colonies were observed on YPDA plus YPDA plates containing drugs plates, however, the size of the** colonies were **significantly smaller than that of the wild-type cells.**

**^☆^indicates that the naming of genes is after the *S. cerevisiae* counterparts as the common name in *S. pombe* is not available.**

**N/A indicates that common gene name is not applicable.**

* **indicates poor growth on YPDA plates.**
