# Supplementary material for: Genome-Wide Screening for Genes Associated with Valproic Acid Sensitivity in Fission Yeast
Source: PLoS One. 2013 Jul 5;8(7):e68738. doi: 10.1371/journal.pone.0068738 (PMC3702616; doi:10.1371/journal.pone.0068738)
Supplement: Table S2 — S. pombe genes identified in the screen for CaCl2-sensitivity in 148 sensitive strains. (DOCX) [file pone.0068738.s007.docx]

**Table S2 CaCl_2_-sensitivity in 148 sensitive strains**

| **Systematic Name** | **Gene Name** | **CaCl_2_ sensitivity** | **Systematic Name** | **Gene Name** | **CaCl_2_ sensitivity** |
| --- | --- | --- | --- | --- | --- |
| VPA- and SB-sensitive strains | | | | | |
| SPBC530.01 | gyp1 | +++ | SPBC119.08 | pmk1 | +++ |
| SPBC4F6.10 | vps901 | +++ | SPBPB2B2.14c | **N/A** | +++ |
| SPCC757.09c | rnc1 | +++ | SPAC3H5.08c | **N/A** | +++ |
| SPBC13E7.08c | leo1^☆^ | +++ | SPAC9E9.14 | vps24 | ++ |
| SPCC11E10.06c | elp4 | ++ | SPAC12B10.03 | bun62 | ++ |
| SPAC1006.03c | red1 | ++ | SPBC609.04 | caf5 | ++ |
| SPAPB1E7.02c | mcl1 | ++ | SPAC19G12.08 | scs7 | ++ |
| SPBC543.07 | pek1 | ++ | SPBC29A10.16c | cyb5^☆^ | ++ |
| SPBC725.09c | hob3 | ++ | SPBC3E7.09 | slp1^☆^ | ++ |
| SPAC22F3.13 | tsc1 | ++ | SPBC31F10.02 | **N/A** | ++ |
| SPBC1289.09 | tim21 | ++ | SPAC25B8.05 | deg1^☆^ | ++ |
| SPBC2G5.06c | hmt2 | ++ | SPBC1778.05c | **N/A** | ++ |
| SPBC6B1.06c | ubp14 | ++ | SPAC6G9.15c | **N/A** | ++ |
| SPBC1105.08 | emp70 | + | SPBC11B10.10c | pht1 | + |
| SPCC31H12.08c | ccr4 | + | SPAC23C4.08 | rho3 | + |
| SPAC20H4.03c | tfs1 | + | SPAC5D6.09c | mug86 | + |
| SPAC29B12.06c | rcd1 | + | SPBC4F6.11c | **N/A** | + |
| SPCC74.02c | **N/A** | + | SPBP4H10.17c | mrps2 | + |
| SPBC13G1.08c | ash2 | + | SPBC3H7.03c | kgd1^☆^ | + |
| VPA-sensitivity strains | | | | | |
| SPBC4F6.12 | pxl1 | +++ | SPBC106.17c | cys2 | +++ |
| SPBC23E6.08 | sat1 | ++ | SPBC16G5.02c | **N/A** | ++ |
| SPBC119.12 | rud3 | ++ | SPBC21D10.10 | bdc1 | ++ |
| SPAC4G9.13c | vps26 | ++ | SPCC895.05 | for3 | ++ |
| SPBC21C3.02c | dep1 | + |  |  |  |

**+++ indicates that the cells completely failed to grow on YPDA plus 0.2 mM CaCl_2_ plates.**

**++ indicates that tiny colonies were observed to grow on YPDA plus 0.2 mM CaCl_2_ plates.**

**+ indicates that colonies were observed on YPDA plus 0.2 mM CaCl_2_ plates, however, the size of the** colonies were **significantly smaller than that of the wild-type cells.**

**^☆^indicates that the naming of genes is after the *S. cerevisiae* counterparts as the common name in *S. pombe* is not available.**

**N/A indicates that common gene name is not applicable.**
